# Identifying research priorities for effective retention strategies in clinical trials

**DOI:** 10.1186/s13063-017-2132-z

**Published:** 2017-08-31

**Authors:** Anna Kearney, Anne Daykin, Alison R. G. Shaw, Athene J. Lane, Jane M. Blazeby, Mike Clarke, Paula Williamson, Carrol Gamble

**Affiliations:** 1 North West Hub for Trials Methodology Research/Clinical Trial Research Centre, Biostatistics, University of Liverpool, Institute of Child Health, Alder Hey NHS Trust, Liverpool, L12 2AP UK; 2 ConDuCT-II Hub for Trials Methodology Research, University of Bristol, Canynge Hall, 39 Whatley Road, Bristol, BS8 2PS UK; 3 Centre for Public Health, Queen’s University of Belfast, University Road, Belfast, BT7 1NN UK; 4 North West Hub for Trials Methodology Research/Clinical Trial Research Centre, Biostatistics, University of Liverpool, Block F Waterhouse Building, 1-5 Brownlow Street, Liverpool, L69 3GL UK

**Keywords:** Missing data, Attrition, Retention, Clinical trials, Missing data strategies, Study design

## Abstract

**Background:**

The failure to retain patients or collect primary-outcome data is a common challenge for trials and reduces the statistical power and potentially introduces bias into the analysis. Identifying strategies to minimise missing data was the second highest methodological research priority in a Delphi survey of the Directors of UK Clinical Trial Units (CTUs) and is important to minimise waste in research. Our aim was to assess the current retention practices within the UK and priorities for future research to evaluate the effectiveness of strategies to reduce attrition.

**Methods:**

Seventy-five chief investigators of NIHR Health Technology Assessment (HTA)-funded trials starting between 2009 and 2012 were surveyed to elicit their awareness about causes of missing data within their trial and recommended practices for improving retention. Forty-seven CTUs registered within the UKCRC network were surveyed separately to identify approaches and strategies being used to mitigate missing data across trials.

Responses from the current practice surveys were used to inform a subsequent two-round Delphi survey with registered CTUs. A consensus list of retention research strategies was produced and ranked by priority.

**Results:**

Fifty out of seventy-five (67%) chief investigators and 33/47 (70%) registered CTUs completed the current practice surveys. Seventy-eight percent of trialists were aware of retention challenges and implemented strategies at trial design. Patient-initiated withdrawal was the most common cause of missing data. Registered CTUs routinely used newsletters, timeline of participant visits, and telephone reminders to mitigate missing data. Whilst 36 out of 59 strategies presented had been formally or informally evaluated, some frequently used strategies, such as site initiation training, have had no research to inform practice.

Thirty-five registered CTUs (74%) participated in the Delphi survey. Research into the effectiveness of site initiation training, frequency of patient contact during a trial, the use of routinely collected data, the frequency and timing of reminders, triggered site training and the time needed to complete questionnaires was deemed critical. Research into the effectiveness of Christmas cards for site staff was not of critical importance.

**Conclusion:**

The surveys of current practices demonstrates that a variety of strategies are being used to mitigate missing data but with little evidence to support their use. Six retention strategies were deemed critically important within the Delphi survey and should be a primary focus of future retention research.

**Electronic supplementary material:**

The online version of this article (doi:10.1186/s13063-017-2132-z) contains supplementary material, which is available to authorized users.

## Background

The challenges of recruiting and retaining participants in clinical trials are well documented [1–4] and addressing these is of critical importance. A recent Delphi survey with directors of UKCRC Clinical Trial Units (CTUs) established that identifying methods to improve recruitment was a top methodological research priority with methods to minimise attrition and the development of core outcome sets as joint second [5].

These priorities are in line with moves to minimise waste in research, ensuring that trials are as robust and cost-effective as possible [6–10]. One of the key ways to achieve this is to maximise the retention of all recruited patients in the study and the collection, analysis and reporting of a complete set of outcomes for them. Whilst there are a number of projects addressing recruitment challenges it is important to ensure there is an equal focus on retention. Recruiting and randomising people who are not subsequently retained for the measurement of the primary outcome may be worse for the analysis of the trial than not randomising that patient at all.

Missing data arises from patients being lost to follow-up or withdrawing before data collection time points, difficulties in measuring and recording outcomes for patients who are retained, incomplete or missing patient-reported outcomes, or by excluding data from randomised patients from the analysis population. Missing primary-outcome data in clinical trials is a common problem which has the potential to reduce the power of the trial and can introduce bias if the reasons or amounts of missing data are different across arms. Both the Food and Drug Administration (FDA) and European Medicines Agency (EMA) guidelines [11–14] strongly emphasise the need to mitigate missing data within trial design and conduct, as statistical methods ‘cannot robustly recover the estimates from a complete data set’.

Our aim is to understand the current practices employed by UK trialists to mitigate missing data in clinical trials. To achieve this we conducted a survey of chief investigators of National Institute of Health Research (NIHR) Health Technology Assessment (HTA)-funded trials and CTUs registered within the UK Clinical Research Collaboration (UKCRC) network. A subsequent Delphi survey across registered CTUs was undertaken to establish retention research priorities to minimise missing data in clinical trials.

## Methods

### Surveys of current practices for mitigating missing data

Two different surveys of HTA chief investigators and registered CTUs were used to capture current retention practices. HTA chief investigators were surveyed to provide a clinical perspective of retention-based strategies used within specific trials representing a range of disease areas. In contrast, registered CTUs were surveyed to identify non-clinical expertise and insights pertinent across a wide range of trial designs. Whilst it is possible some HTA-funded trials engaged the expertise of registered CTUs, the two surveys were designed to elicit responses from the different perspectives.

A cohort of 76 NIHR Health Technology Assessment Programme (HTA)-funded randomised trials were identified through their website portfolio [15]. The NIHR HTA portfolio was chosen as it represents the largest public funder of current and ongoing trials and the start dates ensured at least 1 year’s recruitment following any pilot work or delays obtaining governance approvals. Two-arm, parallel trials were included. Exclusion criteria were pilot studies, patient preference trials, phase 1 and 2 trials, and studies evaluating longer-term follow-up of previous trials and substudies. This cohort was identified as part of a wider project on retention. Parallel trials with more than two arms were also excluded for consistency across linked projects.

Chief investigators were surveyed to identify whether retention concerns were identified or known at the trial outset and which missing data strategies were implemented either during the trial design or subsequently during trial conduct (Additional file 1). Survey questions included the causes of missing data within their current trials and whether observed levels of missing data were higher, lower or as expected compared to their estimations at trial outset. Respondents were also asked to recommend the three most effective retention practices based on their experience of clinical trials.

A second survey was emailed to 47 registered CTUs identified from the UK Clinical Research Collaboration (UKCRC) website in August 2014 (Additional file 2). Surveys were initially sent to registered CTU directors to complete or delegate as appropriate. If there was no response, attempts were made to identify contact details for other senior staff such as deputy directors, operational directors, senior trial managers or senior statisticians. Fifty-nine strategies to reduce missing data were collated from the Cochrane review and the published results of previous surveys [16, 17]. Registered CTUs were asked to identify which of these strategies they had used within their trials either during the initial design or later in response to specific challenges. Registered CTUs also had the opportunity to suggest strategies that they felt were missing. For each strategy selected, registered CTUs were asked whether it was used routinely or occasionally and whether they had evaluated its effectiveness. Evaluations were classified as either a formal nested study or an informal evaluation comparing retention rates before and after implementation. Questions also explored how frequently sample size calculations were adjusted for missing data, what percentage of missing data was used and the justification for this.

Both surveys were created in MS Word and distributed via email to allow collaboration within the relevant trial teams and registered CTUs.

One author (AK) categorised free-text fields and analysed binary responses from the surveys using SPSS 22.

### Delphi survey

A Delphi survey was used to gain consensus amongst the registered CTUs to identify which missing data strategies should be prioritised for future research to evaluate their effectiveness. Registered CTUs were invited to take part in the Delphi survey due to their knowledge across clinical trials so that established priorities would be pertinent to the majority of trials undertaken.

Forty-seven CTU directors, or their proxy from the previous survey, were invited by email to take part in a two-round online Delphi survey. During registration participants were given a unique ID number to allow the survey to be completed across a number of sessions and link responses between rounds.

A list of 67 missing data strategies were compiled for use in round 1 based on the results of the surveys shared with CTUs and HTA chief investigators. Registered CTU responses directed changes to the 59 strategies initially used: three strategies were amended for clarity; four strategies were separated to allow delineation between strategies aimed at sites and participants; and similar strategies were grouped together to minimise Delphi survey burden (Additional file 3: Table S1). Two topics were removed as they were not used by registered CTUs (Additional file 3: Table S2). Twelve new strategies were added, 10 of which were influenced by responses from the chief investigator’s survey. These predominantly focussed on the timing and frequency of strategies, e.g. newsletters, patient contact, site contact and questionnaires and were in added in response to comments in the chief investigator’s survey about ‘regular’ contact.

Participants were invited to score each strategy using a scale of 1–9 based on the GRADE guidelines [18]. Scores 1–3 indicated that research into the effectiveness of the retention strategy was not important, 4–6 indicated that it was important but not critical and 7–9 indicated that research was of critical importance. All survey participants could suggest additional retention strategies during round 1 and could abstain from scoring any strategy.

**Fig. 1:**
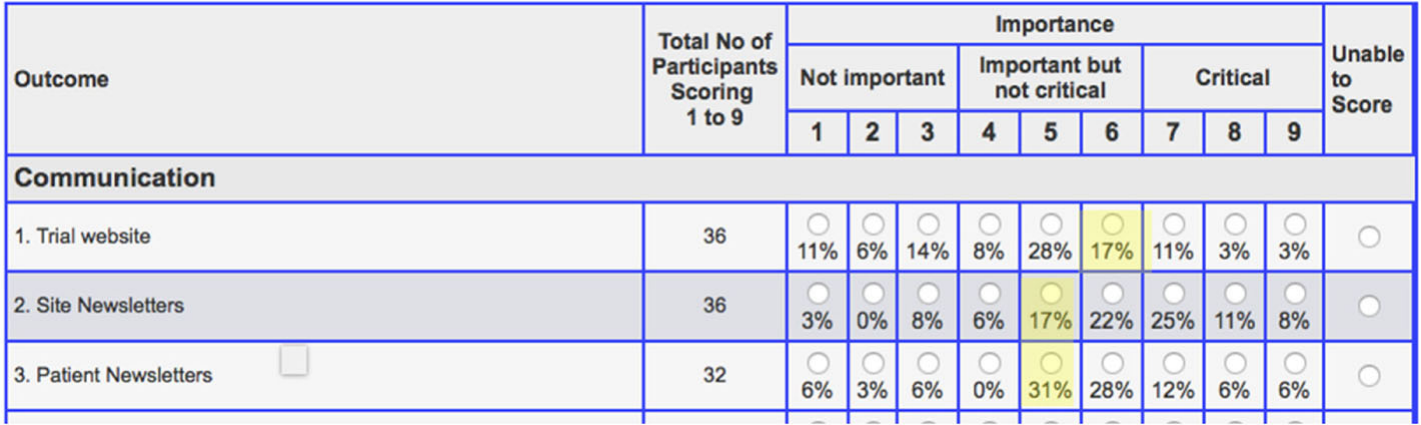
An example of the scoring software used in round 2 of the Delphi survey. Individual participant scores were highlighted in yellow, with percentage of all respondent scores listed beneath each radio button

All participants who completed round 1 were invited to participate in round 2 where they were shown a summary of the group’s scores from the previous round and given the option of changing their individual scores or keeping them the same (Fig. 1). All strategies from round 1 were used in the subsequent round. Additional strategies suggested by participants were reviewed by CG and AK for inclusion in round 2.

Incentive prize draws of £75 and £25 high-street gift vouchers were offered for responses to round 2 within 7 and 12 days, respectively. The aim of the incentive was to improve response rates and minimise the need for reminders. Participants were notified of the incentive within the email invite to round 2.

Consensus was predefined using criteria used in a similar methodological priority setting exercise and core outcome development [5, 19]. Consensus that research was of critical importance was reached if > 70% of scores were 7–9 and < 15% of scores were 1–3. Consensus that research was not important was reached if > 70% of scores were 1–3 as long as < 15% scores were 7–9. All research topics from round 2 were ranked according to the percentage of participants scoring a research topic as critically important (scores 7–9). Where strategies achieved the same score they were then ranked order of the percentage of scores 4–6.

## Results

**Fig. 2:**
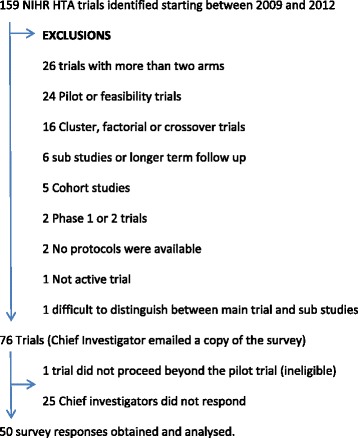
Cohort of National Institute of Health Research (NIHR) Health Technology Assessment Programme (HTA)-funded trials. A search of the NIHR HTA portfolio website was conducted on 23 September 2014 using a keyword ‘randomised’, limited to primary research

Seventy-six chief investigators were approached to complete the survey, but one trial responded to confirm that it did not proceed following the initial pilot and was excluded from the cohort. Fifty of the remaining 75 (67%) surveys were returned (Fig. 2). A breakdown of responder role can be found in Additional file 3: Table S3. Trials represented a broad range of health conditions and intervention types (Additional file 3: Table S4). Over half the trials had closed to recruitment, and 38 (76%) of the trials were aiming to recruit no more than 1000 participants.

Thirty-nine trials (78%) were aware of retention issues at the outset of the trial, with 19 (49%) citing challenges associated with the patient population, such as high mortality or a mobile population, and 16 (41%) were concerned about patients not returning data (Additional file 3: Table S5). Only seven of the 50 trials (14%) stated that current missing data levels were higher than expected at trial design.

Forty-one (84%) trials experienced missing data caused by patient-initiated withdrawal, 30 (61%) through losing contact with patients and 24 (49%) by patients not returning data. However, 14 trials (29%) also reported that data was missing because patients did not attend visits, 12 (25%) due to clinical staff failing to take measurements and 10 (20%) reported that data was not provided by clinical staff (Table 1).

**Table 1:** Current causes of missing data within the cohort of Health Technology Assessment Programme (HTA)-funded trials

Causes of missing data	Number of trials (%) *n* = 49^a^
Patients withdrawing	41 (84%)
Losing contact with patients	30 (61%)
Patients not returning questionnaire	24 (49%)
Patient deaths	23 (47%)
Clinicians withdrawing patients	17 (35%)
Patients not attending a visit/clinic	14 (29%)
Missed measurement by clinical staff	12 (25%)
Patient outcomes other than death preventing measurement, e.g. coma, too ill to complete measures	10 (20%)
Data not provided by clinical staff	10 (20%)
Other	6 (12%)
Technology problems	4 (8%)
Laboratory problems	2 (4%)

Survey respondents chose all causes of missing data observed in their trial.^a^One person did not complete the question

Based on the respondent’s experiences of clinical trials, the most effective practices for mitigating missing data were robust monitoring and working closely with research sites. Twenty-five (50%) trials recommended monitoring practices to identify, track and rigorously follow up missing data, 15 (30%) recommended maintaining good relationships with trial sites ensuring regular contact, 11 (22%) highlighted the importance of site training and 10 (20%) recommended offering multiple approaches to collect data such as home visits or telephone interviews (Table 2).

**Table 2:** Top five recommended practices to mitigate missing data recommended by chief investigators

Retention strategy	Number of respondents (%) *n* = 50
Monitoring (procedures, methods and systems for monitoring data return and following up outstanding data)	25 (50%)
Good site relationship/regular contact with sites to ensure buy in	15 (30%)
Site training (initiation training and triggered training)	11 (22%)
Multiple methods of data collection	10 (20%)
Well-chosen measures and outcomes	6 (12%)

See Additional file 3: Table S7 for complete list of recommend practices

### Survey of registered CTUs

Thirty-three out of forty-seven (70%) registered CTUs responded to the survey of current practices. Twenty-nine (90%) registered CTUs routinely adjust their sample size to account for missing data of which 19 (66%) used evidence from other trials to inform the levels of adjustment, nine (31%) used their own past experience, four (14%) used pilot data, two (7%) used estimated figures from the chief investigator, two (7%) used a standard 20% dropout rate and one (3%) used a best guess (Additional file 3: Table S9).

Newsletters were the most routinely used missing data strategy reported by 23 registered CTUs (70%) (Table 3). A total of 30 registered CTUs reported using newsletters at some point to mitigate missing data, 20 of which used them to communicate with sites, one used them to communicate with patients and nine registered CTUs used them with both audiences.

**Table 3:** Missing data interventions and reported evaluations into their effectiveness (registered Clinical Trial Unit (CTU) survey Questions 6 and 7)

Missing data intervention	Respondents who have used the intervention (% of all survey respondents, *n* = 33)	Used routinely (% of all survey respondents, *n* = 33)	Used occasionally (% of all survey respondents, *n* = 33)	No response for frequency of use (% of respondents who used intervention)	Before/after evaluation (informal)	Nested RCT evaluation (formal)	Total evaluations
Newsletters^a^	30 (91%)	23 (70%)	5 (15%)	2 (7%)	2	2	4
A timeline of participant visits for sites	24 (73%)	19 (58%)	4 (12%)	1 (4%)	0	0	0
Inclusion of prepaid envelope (questionnaires)^b^	28 (85%)^c^	19 (58%)	6 (18%)	1 (4%)	0	1	1
Telephone reminders	28 (85%)	18 (55%)	9 (27%)	1 (4%)	2	1	3
Data collection scheduled with routine care	25 (76%)	18 (55%)	6 (18%)	1 (4%)	1	1	2
Site initiation training on missing data	19 (58%)	18 (55%)	0 (0%)	1 (5%)	0	0	0
Investigator meetings face to face	22 (67%)	17 (52%)	5 (15%)	0 (0%)	0	1	1
Routines site visits by CTU staff	23 (70%)	15 (45%)	8 (24%)	0 (0%)	1	0	1
Targeted recruitment of sites/GPs	21 (64%)	15 (45%)	6 (18%)	0 (0%)	0	2	2
Flexibility in appointment times	21 (64%)	15 (45%)	4 (12%)	2 (10%)	1	1	2
Communication of trial results	20 (61%)	15 (45%)	3 (9%)	2 (10%)	1	0	1
Investigator teleconferences	22 (67%)^d^	15 (45%)	8 (24%)	0 (0%)	0	1	1
Questionnaires completed in clinic^b^	22 (67%)	15 (45%)	5 (15%)	2 (9%)	1	1	2
Minimising frequency of questionnaires^b^	21 (64%)	15 (45%)	3 (9%)	3 (14%)	0	0	0
Short questionnaire^b^	24 (73%)	14 (42%)	9 (27%)	1 (4%)	0	1	1
Collecting multiple contact details	22 (67%)	13 (39%)	8 (24%)	1 (5%)	2	1	3
Email reminders	21 (64%)	13 (39%)	6 (18%)	2 (10%)	4	0	4
Postal reminders	23 (70%)	12 (36%)	10 (30%)	1 (4%)	2	2	4
Total design method for Questionnaires^b^	15 (46%)	12 (36%)	3 (9%)	0 (0%)	1	1	2
Re-imbursement of participant expenses	24 (73%)^e^	11 (33%)	12 (36%)	0 (0%)	1	1	2
Triggered site training on missing data	23 (70%)	11 (33%)	12 (36%)	0 (0%)	0	0	0
Use of routinely collected data	29 (88%)	10 (30%)	17 (52%)	2 (7%)	2	1	3
Contact GPs for missing data/trace patients	27 (82%)	10 (30%)	14 (42%)	3 (11%)	0	1	1
Patient diaries	26 (79%)	10 (30%)	13 (39%)	3 (12%)	1	0	1
Enhanced cover letter (questionnaires)^b^	14 (43%)	9 (27%)	5 (15%)	0 (0%)	1	2	3
Staggered per patient payments to sites	20 (61%)	8 (24%)	12 (36%	0 (0%)	0	0	0
Patient data entry	18 (55%)	8 (24%)	8 (24%)	2 (11%)	0	1	1
Trial identity cards	14 (43%)	8 (24%)	5 (15%)	1 (7%)	0	0	0
Telephone questionnaires	20 (61%)	7 (21%)	11 (33%)	2 (10%)	2	1	3
Trial website	18 (55%)	7 (21%)	10 (30%)	1 (6%)	0	0	0
Taking contact details for a friend/family	13 (40%)	7 (21%)	6 (18%)	0 (0%)	0	1	1
Long but clear questionnaire^b^	10 (31%)	7 (21%)	2 (6%)	1 (10%)	0	0	0
Only collecting the primary outcome for patients with missing data	17 (52%)	6 (18%)	10 (30%)	1 (6%)	2	1	3
Gift	18 (55%)	6 (18%)	10 (30%)	2 (11%)	1	1	2
ONS flagging	16 (49%)^e^	6 (18%)	7 (21%)	2 (13%)	0	0	0
Flexibility in appointment locations	14 (43%)	6 (18%)	6 (18%)	2(14%)	1	0	1
Money/gift voucher given on completion of a milestone	17 (52%)	5 (15%)	12 (36%)	0 (0%)	1	0	1
Contacting patients between visits	13 (40%)	4 (12%)	9 (27%)	0 (0%)	0	0	0
Christmas and birthday cards	13 (40%)	4 (12%)	9 (27%)	0 (0%)	0	1	1
Freephone number for updating contact	7 (22%)	4 (12%)	3 (9%)	0 (0%)	0	0	0
Follow-up through patient notes only	19 (58%)^e^	3 (9%)	15 (45%)	0 (0%)	0	0	0
Transport to and from appointments	8 (25%)	3 (9%)	4 (12%)	1 (13%)	0	0	0
SMS text reminders	16 (49%)^e^	2 (6%)	13 (39%)	0 (0%)	3	1	4
Trial certificate	8 (25%)	2 (6%)	6 (18%)	0 (0%)	0	0	0
Medical questions first in questionnaire^b^	2 (7%)	2 (6%)	0 (0%)	0 (0%)	0	0	0
Generic questions first in questionnaire^b^	3 (10%)	2 (6%)	0 (0%)	1 (33%)	0	0	0
Questionnaires sent less than 3 weeks after a visit^b^	2 (7%)	2 (6%)	0 (0%)	0 (0%)	0	0	0
Other (questionnaires) ^b^	2 (7%)	2 (6%)	0 (0%)	0 (0%)	0	0	0
Money/gift voucher given regardless	8 (25%)	1 (3%)	7 (21%)	0 (0%)	1	2	3
Case management	3 (10%)	1 (3%)	2 (6%)	0 (0%)	0	0	0
Other	2 (7%)	1 (3%)	1 (3%)	0 (0%)	0	0	0
Personal touch (questionnaires)^b^	9 (28%)	1 (3%)	8 (24%)	0 (0%)	0	0	0
Questions about health issue first in questionnaire^b^	1 (4%)	1 (3%)	0 (0%)	0 (0%)	1	0	1
Questionnaires sent before clinic visit^b^	5 (16%)	1 (3%)	4 (12%)	0 (0%)	0	1	1
Prize draw limited to trial participants	6 (19%)	0 (0%)	6 (18%)	0 (0%)	0	0	0
Social media	5 (16%)^e^	0 (0%)	3 (9%)	1 (20%)	0	0	0
Priority or recorded post (questionnaires)^b^	4 (13%)	0 (0%)	3 (9%)	1 (25%)	0	0	0
Crèche service	0 (0%)	0 (0%)	0 (0%)	0 (0%)	0	0	0
Behavioural motivation	0 (0%)	0 (0%)	0 (0%)	0 (0%)	0	0	0
Charity donation	0 (0%)	0 (0%)	0 (0%)	0 (0%)	0	0	0
National lottery ticket or similar public draw	0 (0%)	0 (0%)	0 (0%)	0 (0%)	0	0	0
Total					35	31	66
Number of strategies that have been evaluated (% of all strategies, *n* = 59)					23 (39%)	26 (44%)	36 (61%)

^a^One case where the newsletters were used with patients only, nine cases where newsletters were used with both patients and research sites, and 20 cases where they were only used with research sites. ^b^Strategies used to enhance questionnaire response rates from Question 7 of the registered CTU survey ^c^Two respondent stated that they would not use this intervention again. ^d^One person reported both occasional and routine use. ^e^One respondent stated that they would not use this intervention again

*CTU* Clinical Trial Unit, *GP* general practitioner, *ONS* Office for National Statistics, *RCT* randomised controlled trial, *SMS* short message service

Nineteen registered CTUs (58%) reported routinely using a timeline of participant visits and the inclusion of a prepaid envelope for questionnaires, 18 (55%) regularly used telephone reminders, data collection scheduled with routine care and site initiation training on missing data. Seventeen (52%) of registered CTUs reported occasionally using routinely collected data, 15 (45%) occasionally used follow-up through patient notes, 14 (42%) contacting GPs for missing data/to trace patients, 13 (39%) patient diaries, SMS (text) reminders and re-imbursement of participant expenses. No-one reported using a crèche service, behavioural motivation, charity donations or public draws such as national lottery tickets.

Thirty-one planned, ongoing or complete nested randomised control trials evaluations were reported for 26 out of the 59 (44%) different missing data strategies. Thirty-five informal evaluations of 23 interventions assessed effectiveness by comparing retention before and after the implementation of a retention strategy. In total, 36 of the 59 listed strategies had had some form of evaluation (Table 3). However, no assessments of site initiation training or a timeline of participant visits had been undertaken despite their frequent use by registered CTUs.

### Delphi survey

Thirty-five (74%) registered CTUs responded to round 1 of the Delphi survey with 34 (97%) completing round 2. Two people independently responded on behalf of one registered CTU. Both sets of answers were included and we report the analysis for 36 responses in round 1 and 35 responses in round 2.

In round 1 consensus was reached on two strategies; the use of routinely collected data (72%) and site initiation training on missing data (75%) (Table 4).

**Table 4:** Delphi survey research priorities for assessing the effectiveness of missing data interventions

Scores	Round 1			Round 2			Ranking
Missing data intervention	%1–3	%4–6	%7–9	%1–3	%4–6	%7–9	
Site initiation training on missing data^a^	6%	19%	75%	6%	11%	83%	1
Frequency of patient contact during the trial^a^	3%	31%	66%	0%	21%	79%	2
Use of routinely collected data^a^	6%	22%	72%	6%	17%	77%	3
Frequency and timing of reminders^a^	0%	34%	66%	0%	24%	76%	4
Triggered site training on missing data^a^	6%	31%	64%	3%	23%	74%	5
Length/time needed to complete the questionnaire^a^	11%	28%	61%	9%	17%	74%	6
Frequency of contact between central trial staff and investigators	11%	31%	58%	6%	26%	69%	7
Impact of site recruitment rates on data collection	9%	31%	60%	9%	23%	69%	8
Postal or online questionnaires	3%	39%	58%	3%	31%	66%	9
Frequency of questionnaires	6%	36%	58%	6%	29%	66%	10
Data collection scheduled with routine care	8%	31%	61%	9%	26%	66%	11
ONS flagging of patients	15%	27%	58%	16%	19%	66%	12
Impact of local site researcher/clinical staff continuity	6%	34%	60%	3%	32%	65%	13
Telephone reminders	9%	34%	57%	9%	26%	65%	14
Contacting GPs for missing data or to trace patients	17%	31%	51%	9%	26%	65%	14
Only collecting the primary outcome for patients with missing primary and secondary data	6%	32%	62%	6%	30%	64%	16
Email reminders	0%	45%	55%	3%	34%	63%	17
Patient data entry, e.g. use of mobile phone applications (apps), online data or other systems	9%	34%	57%	6%	32%	62%	18
Staggered per patient payments based on patient progress and data collection	9%	29%	63%	9%	29%	62%	19
A timeline reminder of participant visits for sites	11%	37%	51%	9%	32%	59%	20
Flexibility in appointment times, e.g. data collection window	6%	42%	53%	6%	37%	57%	21
Site selection strategies	11%	37%	51%	11%	31%	57%	22
Questionnaires completed in the presence of researchers/clinical staff	11%	39%	50%	11%	31%	57%	22
Case management, e.g. arranging appointments and helping patients access health care	19%	35%	45%	20%	23%	57%	24
Re-imbursement of participant expenses	11%	37%	51%	6%	38%	56%	25
Postal reminders	11%	40%	49%	9%	35%	56%	26
Questionnaires returned to local sites vs central office, e.g. is monitoring of response rates and follow-up of missing questionnaires best performed by local sites or central trial offices	9%	37%	54%	9%	35%	56%	26
Data collected by phoning the patient	11%	37%	51%	9%	37%	54%	28
Inclusion of prepaid envelope	22%	36%	42%	23%	26%	51%	29
SMS text reminders	12%	45%	42%	13%	38%	50%	30
Teleconference meetings with investigators	11%	42%	47%	9%	43%	49%	31
Clinician/researcher-collected outcomes versus PROMS (patient-reported outcome measures)	NA	NA	NA	13%	41%	47%	32
Location where questionnaires are completed, e.g. home or clinic	8%	42%	50%	9%	46%	46%	33
Retention and withdrawal information within the Patient Information Sheets	14%	42%	44%	14%	40%	46%	34
Follow-up through patient notes only	14%	43%	43%	9%	47%	44%	35
Research nurse teleconferences or face-to-face meetings	NA	NA	NA	3%	54%	43%	36
Use of social media to contact participants	13%	47%	41%	10%	48%	42%	37
Flexibility in appointment locations, e.g. home or clinic	14%	39%	47%	14%	46%	40%	38
Site newsletters	11%	44%	44%	14%	49%	37%	39
Availability of blinded outcome assessors to ensure data availability and quality	NA	NA	NA	29%	35%	35%	40
Routine site visits by CTU staff	8%	53%	39%	3%	63%	34%	41
Timing of sending questionnaires, e.g. before or shortly after a visit	11%	51%	37%	9%	57%	34%	42
Collecting multiple contact details for participants	24%	38%	38%	24%	42%	33%	43
Face-to-face meetings with investigators	6%	58%	36%	6%	63%	31%	44
Patient diaries to collect data	11%	50%	39%	14%	54%	31%	45
Total Design Method (Dillman [31]), a specific approach to maximise questionnaire response rates that utilises cover letters, reminders and resending questionnaires	16%	44%	41%	16%	53%	31%	46
Behavioural motivation strategies, e.g. workshop for patients to help facilitate completion of intervention and follow-up	24%	45%	30%	30%	42%	27%	47
Format of newsletters and mode of delivery	NA	NA	NA	12%	62%	26%	48
Offer of trial results for participants	11%	58%	31%	11%	63%	26%	49
Frequency of newsletters	8%	64%	28%	6%	74%	20%	50
Question order, e.g. health-related, generic or medical questions first	11%	61%	28%	6%	74%	20%	50
Patient newsletters	16%	59%	25%	13%	68%	19%	52
Open trial design	41%	44%	15%	42%	45%	13%	53
Timing of monetary/gift voucher for participants, e.g. given conditionally on completion of assessment or unconditionally at the beginning or end of trial	29%	47%	24%	27%	61%	12%	54
Monetary incentives or gift voucher incentives for participants	29%	44%	26%	30%	58%	12%	55
Transport to and from appointments	14%	63%	23%	15%	76%	9%	56
Taking contact details for friends/family of participants	31%	50%	19%	32%	61%	6%	57
Gift for participant	42%	45%	13%	42%	52%	6%	58
Prize draw limited to trial participants	41%	44%	16%	34%	59%	6%	59
Enhanced cover letter	24%	55%	21%	21%	73%	6%	60
Trial certificate	44%	50%	6%	50%	44%	6%	61
Trial website	31%	53%	17%	26%	69%	6%	62
The use of a Freephone number for updating participant’s contact details	35%	58%	6%	40%	57%	3%	63
Trial identity cards	39%	52%	10%	42%	55%	3%	64
Use of social media to contact site staff	33%	55%	12%	38%	59%	3%	65
Gift for site staff	45%	52%	3%	36%	61%	3%	66
Christmas and/or birthday cards for participants	48%	45%	6%	64%	33%	3%	67
Type of post used, e.g. priority, standard or recorded post	34%	54%	11%	29%	68%	3%	68
Personal touch, e.g. handwritten letter or addition of post it notes	26%	71%	3%	27%	73%	0%	69
Offer of a crèche service	50%	46%	4%	57%	43%	0%	70
Christmas cards for site staff^b^	67%	33%	0%	82%	18%	0%	71

^a^Consensus was achieved that the future research was of critical importance. ^b^Consensus was achieved that future research was not important

*CTU* Clinical Trial Unit, *GP* general practitioner, *NA* not applicable, *ONS* Office for National Statistics, *SMS* short message service

Participants suggested four new strategies during round 1 which were included in round 2: research nurse teleconferences; the use of patient-reported outcome measures (PROMs) versus clinician-collected outcomes; format of newsletters and mode of delivery; availability of blinded outcome assessors to ensure data availability and quality.

Nineteen (54%) of those completing round 2 of the survey responded by the first prize draw deadline, a further nine (26%) by the second prize draw deadline and three (9%) completed the survey after the incentives ended but before the survey closed. A further four (11%) completed the survey after it had officially closed following email or telephone communication but were also included.

In round 2 consensus was reached for seven interventions (Table 4). Consensus criteria for critically important research were met for: site initiation training on missing data; frequency of patient contact during the trial; use of routinely collected data; frequency and timing of reminders; triggered site training on missing data and the length of time needed to complete questionnaires. Research into Christmas and birthday cards for site staff was found to be of low importance by over 70% of respondents.

## Discussion

Surveys of current practices and a Delphi survey have highlighted routinely used approaches within the UK, the lack of evidence informing practice and future methodological research priorities within retention.

A strong focus has been placed on improving recruitment in clinical trials and this is routinely monitored and accepted as a key performance indicator of sites impacting funding received [20]. However, attention needs to be extended to cover the retention of randomised participants with sites monitored and rewarded accordingly. Whilst HTA chief investigators are aware of retention issues and registered CTUs are regularly revising sample sizes and proactively implementing a broad range of strategies to maintain contact with patients, improve questionnaire and data return, minimise patient burden and incentivise patients, retention is currently not used as a key performance indicator within UK research networks and is seldom linked to per-patient payments for research costs.

Whilst survey formats differed, the perspectives of chief investigators and registered CTUs were similar. Chief investigator’s recommendation of good monitoring processes to identify and address any problems with data collection was congruent with registered CTUs’ routine use of strategies that might facilitate this such as telephone reminders and routine site visits. Both registered CTUs and chief investigators also placed a strong emphasis on training and working with local research site staff to minimise missing data, with six of the top 10 strategies routinely used by registered CTUs focussed in this area.

However, many strategies continue to lack evidence for their effectiveness. Whilst 61% (36/59) of strategies had some evaluation reported by registered CTUs, existing evidence was typically low level: 21 strategies had only one nested RCT, and only five strategies had two evaluations that could be combined in a meta-analysis assuming that there was sufficient homogeneity. It is important to replicate findings in multiple trials to ensure the applicability and generalisability and because results of nested RCTs may be more convincing if they span multiple trials.

Many of the research strategies prioritised within the Delphi survey relate to methods routinely used by registered CTUs. Methods all have implications for resource use and despite their frequent use in current practice there was an absence of evidence to support them. Their prioritisation within the Delphi survey likely reflects the desire of registered CTUs to have their practices supported by evidence to ensure that they direct their limited resources to effective methods. More embedded trials (SWATs: Studies Within A Trial) [21] are needed to further the evidence base and avoid wasting resources on unproven and potentially ineffective retention strategies. The MRC-funded START [22] project focusses on SWATs targeting improved recruitment and a similar initiative could be beneficial to improve the evidence base for retention. The results of the Delphi survey provide a list of priorities for future SWATs.

Site initiation training was routinely used within registered CTUs and a top practice recommended by chief investigators, but was one of the strategies with no reported evaluations. It is, therefore, unsurprising that this strategy was identified as the top priority for future methodological research during the Delphi survey. Triggered site training also reached consensus criteria in round 2 reiterating the need to examine how multicentre trials address attrition when patient contact and outcome measurement are often delegated to sites.

Research into site training is supported by the results of recent workshops reviewing recruitment and retention strategies [16, 23]. Anecdotal evidence suggests that coordinated approaches with local sites and strong clinical buy-in can help to retain patient populations perceived to be at risk of attrition [23]. However, analysis of the methods and content of site training have largely focussed on the impact on informed consent and recruitment [24–28], rather than retention of patients and collection of outcome data where there is a paucity of academic literature. Lienard conducted a nested RCT investigating the effect of training and subsequent monitoring visits on data return and quality but found no difference because the study was terminated early [29].

As the Delphi survey topic titles were broad, further work is needed to explore how site training might impact on retention. Chief investigators commented on both content and methods for delivering training within their survey responses. They described using training to communicate data collection priorities and processes, including the opportunity to develop bespoke processes for individual sites. Suggestions of training methods included interactive presentations, newsletters and refresher training. Existing trial processes (e.g. routine use of newsletters, regular contact with sites) may be able to support such site training as well as enhancing or maintaining the buy-in of research site staff. Berger supports this, communicating how training was important in maintaining the continuity of research staff and equipping them to monitor and address withdrawal reasons, negotiate complaints and reiterate the importance of data collection with patients to improve retention [30].

The choice of outcomes for future SWATs are an important consideration for future research. A Cochrane review identified 38 nested randomised studies of retention interventions of which 34 aimed to improve questionnaire return [17]. In our survey of chief investigators, patient withdrawal was the most widely reported cause of missing data and yet there is little published evidence for effective strategies. The four studies measuring patient retention assessed behavioural motivation, case management and a factorial design of a trial certificate and gift. No effect was found and our research shows that these strategies are, at best, infrequently used by registered CTUs.

### Future research

The ranked list of research priorities from the Delphi survey provides a ‘roadmap’ to address uncertainties and systematically evaluate key strategies. Six topics were deemed critically important and should be a primary focus of future retention research.

We strongly recommend that future studies take account of the range of causes of missing data reported by chief investigators and previous studies. Retention strategies applicable to a range of trial designs, such as site training, frequency of patient contact and the frequency and timing of reminders, should assess impact on patient withdrawal, patients lost to follow-up and clinical staff failing to record primary-outcome measurements as well as questionnaire response rates.

Many Delphi survey participants commented on the challenges of scoring the importance of retention strategies when these are often chosen to address challenges within specific trial designs or populations. Considering the number of strategies discussed it was not feasible to explore this and participants were encouraged to score strategies based on the trials frequently delivered within their CTU. Consequently, the results reflect a ranked list of priorities that will have the broadest impact across the range of trials currently undertaken in the UK. Future research is needed to explore the effectiveness of different strategies for specific trial designs, settings and patient populations.

### Strengths and limitations

To our knowledge this is the first study to develop a research agenda for evaluating the effectiveness of retention strategies and so provides a valuable reference point for future methodological work.

A systematic review and surveys of registered CTUs and HTA chief investigators were used to compile the initial list of retention strategies for the Delphi survey. The two different surveys sought to capture both the practical experience of chief investigators within specific trials and the broader experience of registered CTUs that work across multiple trials and different trial designs. Registered CTUs were invited to complete the Delphi survey in order to create a priority list that was applicable to the broad range of trials represented within the UK.

Whilst the study was researcher-led and did not include public or patient perspectives, all three surveys benefited from strong response rates and the Delphi survey retained the majority of participants. Only one person failed to complete round 2 suggesting that the results are unlikely to be affected by attrition bias. The high response rate is attributed to engaging an active network of registered CTUs that were invested in the topic having previously agreed that it was of critical importance [5].

Analysis of the number of retention strategies evaluated by registered CTUs should be considered with some caution as missing responses could not be identified. However, this information did not influence the Delphi process or the ranking of retention strategies for future evaluation.

## Conclusion

Whilst trials are proactively implementing a range of strategies to address retention challenges, further evidence is needed to inform practice and minimise waste on ineffective methods.

The Delphi survey results provide methodological researchers and trialists with a research agenda and a means of effectively channelling resources towards the assessment of retention strategies that impact UK trials. Research should focus on site training, frequency of patient contact, the use of routinely collected data, the frequency and timing of reminders and the length of time needed to complete questionnaires. Research outcomes should reflect the wide range of missing data causes and, in particular, should include an assessment of their impact on patient withdrawal which was reported to be the most common cause of missing data.

## Additional files

Additional file 1: HTA chief investigator survey of current practices. (PDF 742 kb)

Additional file 2: Registered CTU survey of current practices. (PDF 875 kb)

Additional file 3: **Table S1** Compilation of Delphi survey list for round 1. **Table S2** Retention strategies within the initial CTU survey but not included in the subsequent Delphi survey. **Table S3** Respondents to the survey of HTA-funded trials. **Table S4** Contextual data extracted from protocols and survey responses. **Table S5** Anticipated causes of missing data at trial design (Question 5). **Table S6** Differences between the observed and anticipated levels of missing data (Question 8). **Table S7** Effective practices for mitigating missing data recommended from trialists (Question 13). **Table S8** Frequency of CTUs usually adjusting their sample size for missing data (Question 2). **Table S9** What informs the level of attrition adjustment in the sample size calculation? (Question 2). (DOCX 40 kb)

## Abbreviations

CTU: Clinical Trial Unit
HTA: Health Technology Assessment Programme
NIHR: National Institute of Health Research
UK CRC: UK Clinical Research Collaboration
