# Supplementary material for: Identifying research priorities for effective retention strategies in clinical trials
Source: Trials. 2017 Aug 31;18:406. doi: 10.1186/s13063-017-2132-z (PMC5580283; doi:10.1186/s13063-017-2132-z)
Supplement: Supplementary file 1 — HTA chief investigator survey of current practices. (PDF 742 kb) [file 13063_2017_2132_MOESM1_ESM.pdf]

Queen's University Belfast | University of Liverpool | University of Bristol | MRC Medical Research Council

Survey: methods to minimise missing data in randomised trials

Overview: We are contacting you as the Chief Investigator of a HTA funded trial to seek your opinion on effective strategies to minimise missing data in randomised trials. We are also looking at the association between missing data, monitoring approach, trial design characteristics and how outcomes are measured to help inform the development of effective solutions. The results will contribute towards a wider MRC MRP funded project aiming to identify which strategies and practices used to minimise missing data should be assessed in further research.

Definition of missing data: Missing data occurs when randomised participants are not included in the trial analysis for a particular outcome. Missing data may arise due to participant loss to follow up, withdrawal from follow up, failure to measure a particular outcome for a participant or the exclusion of a participant's measured outcome from the analysis.

Confidentiality: Your responses will be treated confidentially. Overall responses and associations will be reported in an anonymous manner.

Completion: The survey takes around 5-10mins to complete. This can be done electronically within this Word document or on paper, depending on your preference. If you are unsure of any trial practice, you could ask your trial manager or supporting CTU to help answer the relevant questions. If your version of Word has problems with the checkboxes, please let us know and we can send an alternate version to you.

Return to: a.kearney@liv.ac.uk by 23rd January 2015

Paper copies can be posted to: Anna Kearney, Clinical Trials Research Centre, University of Liverpool, Institute of Child Health, Alder Hey Children's NHS Foundation Trust, Liverpool, L12 2AP

HTA reference:

Trial title:

1. **When did the above trial open to recruiting and randomising patients?** ______________________________

2. **If applicable, when did the trial close to recruiting and randomising patients?** ________________________

3. **Would the primary outcome have been routinely measured for patients with the relevant condition regardless of their participation in the trial?**

☐	Yes, is a routine care measurement
☐	Is likely to be measured in routine care, but might not be measured depending on local practice
☐	Is unlikely to be measured in routine care, but might be measured depending on local practice
☐	Would not be measured in routine care

Optional comments (for example, if you had more than one primary outcome, or to expand on your answer):

____

____

____

4. **Is the measurement of the primary outcome taken during an appointment that coincides with routine care visits, or is it collected during a trial specific visit?**

☐	Yes, measured during routine care visit
☐	No, measured during a trial specific visit
☐	Varies depending on whether the trial specific visit coincides with routine care visit
☐	Not applicable (primary outcome is not measured at a visit)
☐	Other, specify

5. **When designing the trial were you aware of any factors which made your trial at increased risk of missing data e.g. patient population, technical issues, response rates to surveys etc.?**

☐	Yes (see below)
☐	No (go to Q6)

If yes, what were the factors you were aware of (in particular for your primary outcome) and what did you do to mitigate the risk?

Factor	Strategies used to mitigate the risk of missing data

6. **In addition to any listed above, what strategies have you used to minimise missing data, either to avoid patients withdrawing from the study, or to maximise data collection for patients retained in the trial? This may include strategies you are planning to implement.**

____

____

____

____

____

____

____

7. **Where are levels of missing data reviewed within your trial and how frequently?**

	**How frequently is missing data reviewed? (e.g. never, monthly, 6 monthly, annually)** *Please note we are not interested in the frequency of the meetings or reports, but the frequency with which missing data is reviewed within them.*
Trial Steering Committee meetings	
Independent Data Monitoring Committee meetings	
Trial Management Group meetings	
Internal CTU meetings	
During statistical analysis (e.g. interim reports, periodic monitoring, final report)	
For periodic reports to the sponsor or the funder	
Other, Specify	

8. **How do the observed levels of missing primary outcome data compare with the levels you anticipated at the trial design stage, for example when calculating your original sample size?**

☐	Much higher than expected
☐	Higher than expected
☐	As expected
☐	Lower than expected
☐	Much lower than expected
☐	Other, specify

9. **If you are willing to say, what percentage of missing primary outcome data are you currently observing?** *Please note this answer will not be linked to your trial and will remain confidential.*

____

____

What proportion of this missing data do you think may be obtainable?

____

____

10. **In your opinion why do you think the observed levels of missing data are different to the levels of missing data you predicted at the start of the trial? In your response consider that levels may be higher or lower than expected e.g. a higher or lower questionnaire return rate than anticipated.**

____

____

____

11. Which of the following reasons for missing primary outcome data have occurred in your trial? *(tick all that apply)*

☐	Clinicians have withdrawn patients from treatment and follow-up
☐	Patients have withdrawn from follow up
☐	Losing contact with patients
☐	Patient deaths (unrelated to primary outcome)
☐	Patient outcomes (other than death) which prevent the measurement of the primary outcome
☐	Missed measurements by clinical staff
☐	Data not provided by clinical staff
☐	Failure of patient to return a questionnaire or the material to measure the primary outcome
☐	Failure of patient to attend a visit for the measurement of the primary outcome
☐	Problems with laboratory measurements
☐	Problems with data measured using technology e.g. pedometer, accelerometer, insulin pump etc.
☐	Other, Specify

12. Within your HTA funded trial do you attempt to collect reasons why patients withdraw from trial follow up?

☐	Yes - If so have you had any problems trying to collect reasons (e.g. most patients not willing to say, sites not willing to ask)
☐	No – If so, was there a reason why you have not attempted to collect this information?

13. In your opinion what are the three most effective strategies for minimising missing data that you have used within the trial?

____

____

____

14. If you have any further insights from <u>any</u> trials you have been involved in about effective or ineffective strategies to minimise missing data, please share your thoughts here:

____

____

____

____

____

15. Patient information sheet included:

Lack of patient retention through withdrawal of consent or loss to follow up are key causes of missing data within trials. As part of our work identifying strategies that might mitigate missing data we are interested to understand how withdrawal, outcome data collection and patient retention are communicated to participants. To do this we would like to review the content of patient information sheets from a number of trials, including your HTA funded trial (listed above).

It would be of great help to our research if you (or a colleague) could send us a copy of the trial's Patient Information sheet and consent forms. (For trials recruiting children, please include both the parental and patient information sheets and consent/assent forms)

☐	Yes attached with the completed survey
☐	Please email ____ to obtain a copy.

Many thanks for taking the time to complete this survey.

It would be very helpful to know who completed the survey and if you would be willing to be contacted if we have any further questions about any of the information supplied.

**Name:**	**Date:**
**Role:**	
☐	I am happy to be contacted if you have any further questions about the information I have supplied
☐	I would like a copy of the results when available
