# Supplementary material for: Identifying research priorities for effective retention strategies in clinical trials
Source: Trials. 2017 Aug 31;18:406. doi: 10.1186/s13063-017-2132-z (PMC5580283; doi:10.1186/s13063-017-2132-z)
Supplement: Supplementary file 2 — Registered CTU survey of current practices. (PDF 875 kb) [file 13063_2017_2132_MOESM2_ESM.pdf]

Survey: Methods to minimise missing data in randomised control trials

Overview: This short survey builds on knowledge gained from previous CTU surveys and will contribute towards a wider MRC MRP funded project aiming to identify which strategies and practices used to minimise missing data should be formally evaluated. We will ask about the general approach used within your CTU related to targeting of participants, site staff, and CTU processes in order to reduce missing data.

Definition of missing data: Missing data may arise within randomised control trials due to participant loss to follow up, participant withdrawal or the particular outcome was not measured for a participant or the measurement was not included in the analysis.

Completion: Please pass this survey onto the person/s within your CTU who have an overview of all your trials. It can be completed within the word document or in a printed paper format, depending on your preference.

Return to: a.kearney@liv.ac.uk by Friday 12th September.

Paper copies can be posted to: Anna Kearney, Clinical Trials Research Centre, University of Liverpool, Institute of Child Health, Alder Hey Children's NHS Foundation Trust, Liverpool, L12 2AP

Thank you!

---

1. **What are your considerations when determining an approach to monitoring missing data?**

____

____

____

2. **Do you usually adjust your sample size calculation for missing outcome data at the design stage?**

☐ Yes

If Yes, What level do you generally use? ____________________

Do you provide justification for the level used and if so what informs the level used?

____

____

____

☐ No

3. **What processes does your CTU usually use to reduce missing data?** *(Tick all that apply)*

☐ Remote data capture with real time flagging of missing data items sent to local research site
☐ Central data entry with real time flagging of missing data within the CTU
☐ Automated alerts to trial coordinator of upcoming follow up visits
☐ Use of PPI in trial design or to address missing data issues raised within a trial
☐ Other- Specify ______________________________________

4. What problems have you experienced in requesting data on the reason behind a participant's decision to withdraw from a study? *(Tick all that apply)*

☐ Gaining favourable ethical opinion to ask
☐ Willingness of site staff to ask
☐ No problems experienced
☐ Other- specify ____

5. Which factors should be included when determining the following?*(Tick all that apply)*

	Screening	Recruitment	Retention for duration of trial	Resolution of data queries	Availability of primary outcome data	Other (Optional Comments)
Site performance	☐	☐	☐	☐	☐	
Funders assessment of performance	☐	☐	☐	☐	☐	
Per patient payments for sites	☐	☐	☐	☐	☐	

6. Which methods/practices have you used to minimise patient attrition and missing data from retained patients (excluding practices associated with questionnaire design)?

A list of methods and practices used to minimise missing data has been compiled using Valerie Brueton's 2014 Cochrane Systematic review of missing data interventions and the results of two recent surveys distributed through the CTU network and the Trial Managers Network.

In the subsequent tables please identify which strategies your CTU has used to minimise attrition or missing data by ticking the shaded box in the first column. If your CTU has used any of the strategies for reasons not associated with missing data, please do not tick the box. For all strategies that you have used to address missing data please complete the subsequent columns: who the strategy is aimed at; how regularly the strategy is used; what it is used to address; if there were implementation problems; why it is/ was used; and whether you have evaluated the effectiveness. There is also space for optional comments if you wish to give additional information on the specifics of the strategy, its implementation or evaluation.

Within the table you will be asked to indicate if you are willing to provide an abstract for any formal or information evaluations of missing data strategies. Feedback from the recent Trial Managers survey suggested that more could be done to share practice and knowledge of successful and unsuccessful missing data interventions. Across CTUs there will be a wealth of knowledge as a result of nested RCT and before/ after comparisons which may not always be published. We would like to create a publically available, searchable website of study abstracts evaluating strategies as a reference point for CTUs.

To avoid delays in returning these survey results, we plan to contact you at a later date to collect short abstracts (approx. 300 words) for any completed or ongoing studies. If you are willing to supply an abstract please tick in the right hand grey box against the relevant strategy and provide a contact email when returning the questionnaire.

Please complete the tables on the following pages:

Strategies used by your CTU to minimise missing data

Instructions: For each strategy used by your CTU to minimise missing data please tick the first column and then answer the questions in subsequent columns. NB. If your CTU has used any of the strategies for reasons not associated with missing data there is no need to tick the box or complete subsequent columns

		**Aimed at?** *(Tick all that apply)*		**How regularly is it used?**			**Used to address?** *(Tick all that apply)*		**Problems with implementation?** *(Tick all that apply)* None (X) Resources - finances & staff time (R) Sites reluctant (S) Patients reluctant (P) Not practical in practice (N) Other (O)						**Reason for using?**				**Complete, ongoing or planned evaluations?** *(Tick all that apply)*			**Optional Comments** Space for you to give additional information on the strategy or its application or evaluation. If completing a paper copy please use extra pages as required
	**Strategy used**	Participants	Sites	Would not use again	Used occasionally	Used routinely	Patient attrition	Missing data	**X**	**R**	**S**	**P**	**N**	**O**	Logic	Experience	Best practice	Other (add in comments)	Before/after comparison	Nested RCT	Willing to send abstract	
**Communication Strategies**																						
Trial website	☐	☐	☐	☐	☐	☐	☐	☐	☐	☐	☐	☐	☐	☐	☐	☐	☐	☐	☐	☐	☐	
Newsletters	☐	☐	☐	☐	☐	☐	☐	☐	☐	☐	☐	☐	☐	☐	☐	☐	☐	☐	☐	☐	☐	
Social Media e.g. twitter, facebook, blogs	☐	☐	☐	☐	☐	☐	☐	☐	☐	☐	☐	☐	☐	☐	☐	☐	☐	☐	☐	☐	☐	
Collecting multiple contact details for the patient e.g. home, work, mobile phone numbers	☐	☐	☐	☐	☐	☐	☐	☐	☐	☐	☐	☐	☐	☐	☐	☐	☐	☐	☐	☐	☐	
Taking contact details for a friend/family member	☐	☐	☐	☐	☐	☐	☐	☐	☐	☐	☐	☐	☐	☐	☐	☐	☐	☐	☐	☐	☐	
Free phone no. for updating contact details	☐	☐	☐	☐	☐	☐	☐	☐	☐	☐	☐	☐	☐	☐	☐	☐	☐	☐	☐	☐	☐	
Contacting patients between trial visits	☐	☐	☐	☐	☐	☐	☐	☐	☐	☐	☐	☐	☐	☐	☐	☐	☐	☐	☐	☐	☐	
Christmas and birthday cards	☐	☐	☐	☐	☐	☐	☐	☐	☐	☐	☐	☐	☐	☐	☐	☐	☐	☐	☐	☐	☐	
Communication of trial findings/ results	☐	☐	☐	☐	☐	☐	☐	☐	☐	☐	☐	☐	☐	☐	☐	☐	☐	☐	☐	☐	☐	
Other, Specify ______________________	☐	☐	☐	☐	☐	☐	☐	☐	☐	☐	☐	☐	☐	☐	☐	☐	☐	☐	☐	☐	☐	
**Reminders**																						
Telephone reminders	☐	☐	☐	☐	☐	☐	☐	☐	☐	☐	☐	☐	☐	☐	☐	☐	☐	☐	☐	☐	☐	
Postal reminders	☐	☐	☐	☐	☐	☐	☐	☐	☐	☐	☐	☐	☐	☐	☐	☐	☐	☐	☐	☐	☐	
Email reminders	☐	☐	☐	☐	☐	☐	☐	☐	☐	☐	☐	☐	☐	☐	☐	☐	☐	☐	☐	☐	☐	
SMS Text reminders	☐	☐	☐	☐	☐	☐	☐	☐	☐	☐	☐	☐	☐	☐	☐	☐	☐	☐	☐	☐	☐	
Other, Specify ______________________	☐	☐	☐	☐	☐	☐	☐	☐	☐	☐	☐	☐	☐	☐	☐	☐	☐	☐	☐	☐	☐	

Strategies used by your CTU to minimise missing data

Instructions: For each strategy used by your CTU to minimise missing data please tick the first column and then answer the questions in subsequent columns. NB. If your CTU has used any of the strategies for reasons not associated with missing data there is no need to tick the box or complete subsequent columns

Problems with implementation? (Tick all that apply): None (X), Resources -finances & staff time (R), Sites reluctant (S), Patients reluctant (P), Not practical in practice (N), Other (O)

Optional Comments: Space for you to give additional information on the strategy or its application or evaluation. If completing a paper copy please use extra pages as required

		**Aimed at?** *(Tick all that apply)*		**How regularly is it used?**			**Used to address?** *(Tick all that apply)*		**Problems with implementation?** *(Tick all that apply)*						**Reason for using?**				**Complete, ongoing or planned evaluations?** *(Tick all that apply)*			**Optional Comments**
	**Strategy used**	Participants	Sites	Would not use again	Used occasionally	Used routinely	Patient attrition	Missing data	**X**	**R**	**S**	**P**	**N**	**O**	Logic	Experience	Best practice	Other (add in comments)	Before/after comparison	Nested RCT	Willing to send abstract	
**Making participation convenient**																						
Flexibility in appointment times	☐	☐	☐	☐	☐	☐	☐	☐	☐	☐	☐	☐	☐	☐	☐	☐	☐	☐	☐	☐	☐	
Flexibility in appointment locations	☐	☐	☐	☐	☐	☐	☐	☐	☐	☐	☐	☐	☐	☐	☐	☐	☐	☐	☐	☐	☐	
Re-imbursement of participant expenses	☐	☐	☐	☐	☐	☐	☐	☐	☐	☐	☐	☐	☐	☐	☐	☐	☐	☐	☐	☐	☐	
Case management e.g. helping patients book/ attend appointments, access health info etc.	☐	☐	☐	☐	☐	☐	☐	☐	☐	☐	☐	☐	☐	☐	☐	☐	☐	☐	☐	☐	☐	
Crèche service	☐	☐	☐	☐	☐	☐	☐	☐	☐	☐	☐	☐	☐	☐	☐	☐	☐	☐	☐	☐	☐	
Transport to and from appointments	☐	☐	☐	☐	☐	☐	☐	☐	☐	☐	☐	☐	☐	☐	☐	☐	☐	☐	☐	☐	☐	
Other, Specify ______________________	☐	☐	☐	☐	☐	☐	☐	☐	☐	☐	☐	☐	☐	☐	☐	☐	☐	☐	☐	☐	☐	
**Methods to enhance data collection**																						
Patient diaries	☐	☐	☐	☐	☐	☐	☐	☐	☐	☐	☐	☐	☐	☐	☐	☐	☐	☐	☐	☐	☐	
Trial identity cards	☐	☐	☐	☐	☐	☐	☐	☐	☐	☐	☐	☐	☐	☐	☐	☐	☐	☐	☐	☐	☐	
Behavioural Motivation session/s for patients	☐	☐	☐	☐	☐	☐	☐	☐	☐	☐	☐	☐	☐	☐	☐	☐	☐	☐	☐	☐	☐	
Data collection scheduled with routine care	☐	☐	☐	☐	☐	☐	☐	☐	☐	☐	☐	☐	☐	☐	☐	☐	☐	☐	☐	☐	☐	
Patient data entry systems e.g. web data, mobile applications	☐	☐	☐	☐	☐	☐	☐	☐	☐	☐	☐	☐	☐	☐	☐	☐	☐	☐	☐	☐	☐	
Phone questionnaires	☐	☐	☐	☐	☐	☐	☐	☐	☐	☐	☐	☐	☐	☐	☐	☐	☐	☐	☐	☐	☐	
Contact GPs for missing data /trace patients	☐	☐	☐	☐	☐	☐	☐	☐	☐	☐	☐	☐	☐	☐	☐	☐	☐	☐	☐	☐	☐	
Use of routinely collected data- ONS/HES/ISD	☐	☐	☐	☐	☐	☐	☐	☐	☐	☐	☐	☐	☐	☐	☐	☐	☐	☐	☐	☐	☐	
Follow up through patient notes only	☐	☐	☐	☐	☐	☐	☐	☐	☐	☐	☐	☐	☐	☐	☐	☐	☐	☐	☐	☐	☐	
Only collecting primary outcome for patients who have missing primary and secondary outcome data	☐	☐	☐	☐	☐	☐	☐	☐	☐	☐	☐	☐	☐	☐	☐	☐	☐	☐	☐	☐	☐	
Other, Specify ______________________	☐	☐	☐	☐	☐	☐	☐	☐	☐	☐	☐	☐	☐	☐	☐	☐	☐	☐	☐	☐	☐	

Strategies used by your CTU to minimise missing data

Instructions: For each strategy used by your CTU to minimise missing data please tick the first column and then answer the questions in subsequent columns. NB. If your CTU has used any of the strategies for reasons not associated with missing data there is no need to tick the box or complete subsequent columns

		Aimed at? *(Tick all that apply)*		How regularly is it used?			Used to address? *(Tick all that apply)*		Problems with implementation? *(Tick all that apply)* None (X) Resources - finances & staff time (R) Sites reluctant (S) Patients reluctant (P) Not practical in practice (N) Other (O)						Reason for using?				Complete, ongoing or planned evaluations? *(Tick all that apply)*			Optional Comments: Space for you to give additional information on the strategy or its application or evaluation. If completing a paper copy please use extra pages as required
	**Strategy used**	Participants	Sites	Would not use again	Used occasionally	Used routinely	Patient attrition	Missing data	**X**	**R**	**S**	**P**	**N**	**O**	Logic	Experience	Best practice	Other (add in comments)	Before/after comparison	Nested RCT	Willing to send abstract	
**Incentives**																						
Money/gift voucher given to participants for completion of a milestone e.g. clinic attendance /data return	☐	☐	☐	☐	☐	☐	☐	☐	☐	☐	☐	☐	☐	☐	☐	☐	☐	☐	☐	☐	☐	
Money/gift voucher given to participants regardless of clinic attendance or data return	☐	☐	☐	☐	☐	☐	☐	☐	☐	☐	☐	☐	☐	☐	☐	☐	☐	☐	☐	☐	☐	
Prize draw limited to trial participants	☐	☐	☐	☐	☐	☐	☐	☐	☐	☐	☐	☐	☐	☐	☐	☐	☐	☐	☐	☐	☐	
Charity donation	☐	☐	☐	☐	☐	☐	☐	☐	☐	☐	☐	☐	☐	☐	☐	☐	☐	☐	☐	☐	☐	
Gift e.g. mug, pen, toy	☐	☐	☐	☐	☐	☐	☐	☐	☐	☐	☐	☐	☐	☐	☐	☐	☐	☐	☐	☐	☐	
National Lottery ticket or similar public draw	☐	☐	☐	☐	☐	☐	☐	☐	☐	☐	☐	☐	☐	☐	☐	☐	☐	☐	☐	☐	☐	
Trial certificate	☐	☐	☐	☐	☐	☐	☐	☐	☐	☐	☐	☐	☐	☐	☐	☐	☐	☐	☐	☐	☐	
Other, Specify ______________________________	☐	☐	☐	☐	☐	☐	☐	☐	☐	☐	☐	☐	☐	☐	☐	☐	☐	☐	☐	☐	☐	
**Working with clinical staff at sites**																						
ONS flagging of trial participants	☐	☐	☐	☐	☐	☐	☐	☐	☐	☐	☐	☐	☐	☐	☐	☐	☐	☐	☐	☐	☐	
Site initiation training on missing data	☐	☐	☐	☐	☐	☐	☐	☐	☐	☐	☐	☐	☐	☐	☐	☐	☐	☐	☐	☐	☐	
Triggered site training on missing data	☐	☐	☐	☐	☐	☐	☐	☐	☐	☐	☐	☐	☐	☐	☐	☐	☐	☐	☐	☐	☐	
Staggered per patient payments to sites	☐	☐	☐	☐	☐	☐	☐	☐	☐	☐	☐	☐	☐	☐	☐	☐	☐	☐	☐	☐	☐	
Targeted recruitment of sites/ GPs	☐	☐	☐	☐	☐	☐	☐	☐	☐	☐	☐	☐	☐	☐	☐	☐	☐	☐	☐	☐	☐	
Investigator meetings face to face	☐	☐	☐	☐	☐	☐	☐	☐	☐	☐	☐	☐	☐	☐	☐	☐	☐	☐	☐	☐	☐	
Investigator teleconferences	☐	☐	☐	☐	☐	☐	☐	☐	☐	☐	☐	☐	☐	☐	☐	☐	☐	☐	☐	☐	☐	
Routine site visits by CTU staff	☐	☐	☐	☐	☐	☐	☐	☐	☐	☐	☐	☐	☐	☐	☐	☐	☐	☐	☐	☐	☐	
A timeline of participant visits to help sites remember when visits occur and what data should be collected	☐	☐	☐	☐	☐	☐	☐	☐	☐	☐	☐	☐	☐	☐	☐	☐	☐	☐	☐	☐	☐	
Other, Specify ______________________________	☐	☐	☐	☐	☐	☐	☐	☐	☐	☐	☐	☐	☐	☐	☐	☐	☐	☐	☐	☐	☐	

7. Which questionnaire design/delivery features have you used to minimise missing data?

Please tick which strategies you have used to minimise missing data. <u>For all strategies that you have used</u> please complete the subsequent columns: how regularly the strategy is used; what it is used to address; any problems with implementation; reason for using; and any evaluations of the effectiveness of the strategy.

		How regularly is it used?			Used to address? (Tick all that apply)		Problems with implementation? (Tick all that apply) None (X) Resources -finances & staff time (R) Sites reluctant (S) Patients reluctant (P) Not practical in practice (N) Other (O)						Reason for using?				Complete, ongoing or planned evaluations? (Tick all that apply)			Optional Comments Space for you to give additional information on the strategy or its application or evaluation. If completing a paper copy please use extra pages as required
	**Strategy used**	Would not use again	Used occasionally	Used routinely	Patient attrition	Missing data	**X**	**R**	**S**	**P**	**N**	**O**	Logic	Experience	Best practice	Other (add in comments)	Before/after comparison	Nested RCT	Willing to send abstract	
**Incentives**																				
Enhanced cover letter	☐	☐	☐	☐	☐	☐	☐	☐	☐	☐	☐	☐	☐	☐	☐	☐	☐	☐	☐	
Total Design method (cover letter, return envelope, postcard reminders and replacement questionnaire)*	☐	☐	☐	☐	☐	☐	☐	☐	☐	☐	☐	☐	☐	☐	☐	☐	☐	☐	☐	
Priority or recorded post	☐	☐	☐	☐	☐	☐	☐	☐	☐	☐	☐	☐	☐	☐	☐	☐	☐	☐	☐	
'Personal touch' e.g. handwritten envelope	☐	☐	☐	☐	☐	☐	☐	☐	☐	☐	☐	☐	☐	☐	☐	☐	☐	☐	☐	
Inclusion of prepaid envelope	☐	☐	☐	☐	☐	☐	☐	☐	☐	☐	☐	☐	☐	☐	☐	☐	☐	☐	☐	
Short questionnaire	☐	☐	☐	☐	☐	☐	☐	☐	☐	☐	☐	☐	☐	☐	☐	☐	☐	☐	☐	
Long but clear questionnaire	☐	☐	☐	☐	☐	☐	☐	☐	☐	☐	☐	☐	☐	☐	☐	☐	☐	☐	☐	
Medical questions first	☐	☐	☐	☐	☐	☐	☐	☐	☐	☐	☐	☐	☐	☐	☐	☐	☐	☐	☐	
Generic questions first	☐	☐	☐	☐	☐	☐	☐	☐	☐	☐	☐	☐	☐	☐	☐	☐	☐	☐	☐	
Questions about the health issue first e.g. mental health before alcohol consumption	☐	☐	☐	☐	☐	☐	☐	☐	☐	☐	☐	☐	☐	☐	☐	☐	☐	☐	☐	
Questionnaires sent <3 weeks after a visit	☐	☐	☐	☐	☐	☐	☐	☐	☐	☐	☐	☐	☐	☐	☐	☐	☐	☐	☐	
Questionnaires sent before clinic visit	☐	☐	☐	☐	☐	☐	☐	☐	☐	☐	☐	☐	☐	☐	☐	☐	☐	☐	☐	
Questionnaires completed in clinics	☐	☐	☐	☐	☐	☐	☐	☐	☐	☐	☐	☐	☐	☐	☐	☐	☐	☐	☐	
Minimising frequency of questionnaires	☐	☐	☐	☐	☐	☐	☐	☐	☐	☐	☐	☐	☐	☐	☐	☐	☐	☐	☐	
Other, Specify ________________________________	☐	☐	☐	☐	☐	☐	☐	☐	☐	☐	☐	☐	☐	☐	☐	☐	☐	☐	☐	

* Dillman, Don A. *Mail and telephone surveys*. Vol. 3. Wiley Interscience, 1978.
