# Supplementary material for: Identifying research priorities for effective retention strategies in clinical trials
Source: Trials. 2017 Aug 31;18:406. doi: 10.1186/s13063-017-2132-z (PMC5580283; doi:10.1186/s13063-017-2132-z)
Supplement: Supplementary file 3 — Table S1 Compilation of Delphi survey list for round 1. Table S2 Retention strategies within the initial CTU survey but not included in the subsequent Delphi survey. Table S3 Respondents to the survey of HTA-funded trials. Table S4 Contextual data extracted from protocols and survey responses. Table S5 Anticipated causes of missing data at trial design (Question 5). Table S6 Differences between the observed and anticipated levels of missing data (Question 8). Table S7 Effective practices for mitigating missing data recommended from trialists (Question 13). Table S8 Frequency of CTUs usually adjusting their sample size for missing data (Question 2). Table S9 What informs the level of attrition adjustment in the sample size calculation? (Question 2). (DOCX 40 kb) [file 13063_2017_2132_MOESM3_ESM.docx]

**Identifying effective retention strategies: A research agenda**

Supplementary File Contents

[Survey of HTA funded trials: Result tables 2](#_Toc467058191)

[CTU Survey: Results Tables 12](#_Toc467058192)

## Compilation of Delphi Survey list for Round 1:

| **Delphi Category** | **Delphi question order** | **Retention strategy** | **Changes made from Initial CTU survey** | **Notes** |
| --- | --- | --- | --- | --- |
| **Enhancing data collection** | 40 | Data collection by phoning patients | Amended | Broadened from phone questionnaires |
| **Working with local site staff and monitoring** | 48 | Site selection strategies | Amended | Broadened for clarification from targeted recruitment of sites/ GP’s |
| **Questionnaire specific strategies** | 63 | Location where questionnaires are completed *e.g. home, clinic* | Amended | Broadened from questionnaires completed in clinic to location of completion |
| **Incentives** | 21 | Timing of Monetary /gift voucher incentives for participants *e.g. given conditionally on completion of assessments or unconditionally at the beginning or end of trial* | Grouped | Grouped the two different timing of incentives (unconditional and conditional) |
| **Questionnaire specific strategies** | 60 | Length / time needed to complete questionnaire | Grouped | Grouping of short and long questionnaires. Similar strategies were grouped to keep the Delphi shorter and minimise survey burden and completion times |
| **Questionnaire specific strategies** | 61 | Question order *e.g. health, generic or medical questions first* | Grouped | Grouping of three strategies. Generic questions first, Health questions first and medical questions first. Similar strategies were grouped to keep the Delphi shorter and minimise survey burden and completion times |
| **Questionnaire specific strategies** | 62 | Timing of sending questionnaires *e.g. shortly after a visit, or before a clinic visit* | Grouped | Grouping of questionnaires sent <3 weeks after a visit and questionnaires sent before clinic visit. Similar strategies were grouped to keep the Delphi shorter and minimise survey burden and completion times |
| **Communication** | 4 | Frequency of newsletters | New strategy added | Added based on CI comments about 'regular contact' with sites and patients and in response to the routine use of Newsletters by CTUs |
| **Communication** | 10 | Frequency of patient contact during the trial | New strategy added | Added based on CI comments about 'regular contact' with sites and patients |
| **Communication** | 14 | Retention and withdrawal information within the Patient information sheets | New strategy added | Added based on a comment within the CI survey about the need to have clear communication at consent about what the trial will involve. Also takes into account a CI recommended practice of informing patients of the importance of data with patients. |
| **Reminders** | 19 | Frequency and timing of reminders | New strategy added | Added based on CI comments about 'regular contact' with sites and patients and monitoring processes. |
| **Facilitating trial participation for patients** | 34 | Open trial design | New strategy added | Added by authors |
| **Working with local site staff and monitoring** | 51 | Frequency of contact between central trial staff and local Investigators | New strategy added | Added based on CI comments about 'regular contact' with sites and patients |
| **Working with local site staff and monitoring** | 52 | Impact of local site researcher / clinical staff continuity | New strategy added | Added based on CI comments about 'regular contact' with sites and patients |
| **Questionnaire specific strategies** | 64 | Postal or online questionnaires | New strategy added | Added by authors, but also took into account CI recommended practices of using multiple methods for data collection. |
| **Questionnaire specific strategies** | 65 | Frequency of questionnaires | New strategy added | Added by authors, following on the theme within other new strategies, exploring the frequency of contact/ data collection. |
| **Questionnaire specific strategies** | 66 | Questionnaires completed in presence of a researcher/ clinical staff | New strategy added | Added by authors but was linked to topic 67 (see below) and CI questions over the role of site staff on data collection and return. |
| **Questionnaire specific strategies** | 67 | Questionnaires returned to sites vs to a central trial office *e.g. Is monitoring or response rates and follow up of missing questionnaires best performed by local sites or central trial offices.* | New strategy added | Added based on monitoring comments and the suggestions of central data return within CI’s recommended practices. |
| **Working with local site staff and monitoring** | 54 | Impact of site recruitment rates on data collection | New strategy added | Added by authors |
| **Communication** | 2 | Site Newsletters | Split | Original strategy from CTU survey has been split to take into account the use of the strategy for sites and patients |
| **Communication** | 3 | Patient Newsletters | Split | Original strategy from CTU survey has been split to take into account the use of the strategy for sites and patients |
| **Communication** | 5 | Use of social media to contact participants | Split | Original strategy from CTU survey has been split to take into account the use of the strategy for sites and patients |
| **Communication** | 6 | Use of social media to contact site staff | Split | Original strategy from CTU survey has been split to take into account the use of the strategy for sites and patients |
| **Communication** | 11 | Christmas cards for site staff | Split | Original strategy from CTU survey has been split to take into account the use of the strategy for sites and patients |
| **Communication** | 12 | Christmas and/ or birthday cards for participants | Split | Original strategy from CTU survey has been split to take into account the use of the strategy for sites and patients |
| **Incentives** | 23 | Gift for participants | Split | Original strategy from CTU survey has been split to take into account the use of the strategy for sites and patients |
| **Incentives** | 24 | Gift for site staff | Split | Original strategy from CTU survey has been split to take into account the use of the strategy for sites and patients |
| **Facilitating trial participation for patients** | 32 | Follow up through patient notes only | original |  |
| **Communication** | 1 | Trial Website | original |  |
| **Communication** | 7 | Collecting multiple contact details for participants | original |  |
| **Communication** | 8 | Taking contact details for friends/family of participants | original |  |
| **Communication** | 9 | The use of a Freephone number for updating participant’s contact details | original |  |
| **Communication** | 13 | Offer of trial results for participants | original |  |
| **Reminders** | 15 | Telephone reminders | original |  |
| **Reminders** | 16 | Postal reminders | original |  |
| **Reminders** | 17 | Email reminders | original |  |
| **Reminders** | 18 | SMS text reminders | original |  |
| **Incentives** | 20 | Monetary or gift voucher incentives for participants | original |  |
| **Incentives** | 22 | Prize draw limited to trial participants | original |  |
| **Incentives** | 25 | Trial certificate for participants | original |  |
| **Facilitating trial participation for patients** | 26 | Flexibility in appointment times *e.g. data collection windows* | original |  |
| **Facilitating trial participation for patients** | 27 | Flexibility in appointment locations *e.g. home or clinic* | original |  |
| **Facilitating trial participation for patients** | 28 | Re-imbursement of participant expenses | original |  |
| **Facilitating trial participation for patients** | 29 | Case management *e.g. arranging appointments and helping patient’s access healthcare* | original |  |
| **Facilitating trial participation for patients** | 30 | Offer of a Crèche service | original |  |
| **Facilitating trial participation for patients** | 31 | Transport to and from appointments | original |  |
| **Facilitating trial participation for patients** | 33 | Trial identity cards | original |  |
| **Enhancing data collection** | 35 | Patient diaries to collect data | original |  |
| **Enhancing data collection** | 36 | Behavioural motivation *e.g. workshop for patients to help facilitate completion of intervention and follow up* | original |  |
| **Enhancing data collection** | 37 | Data collection scheduled with routine care | original |  |
| **Enhancing data collection** | 38 | Use of routinely collected data | original |  |
| **Enhancing data collection** | 39 | Patient data entry. *e.g. using Mobile phone applications (apps), online data or other systems* | original |  |
| **Enhancing data collection** | 41 | Contacting GP's for missing data/ tracing patients | original |  |
| **Enhancing data collection** | 42 | ONS flagging of patients | original |  |
| **Enhancing data collection** | 43 | Only collecting the primary outcome for patients with missing data *e.g. prioritising primary outcome data* | original |  |
| **Working with local site staff and monitoring** | 44 | Site initiation training on missing data | original |  |
| **Working with local site staff and monitoring** | 45 | Triggered site training on missing data | original |  |
| **Working with local site staff and monitoring** | 46 | Routine site visits by CTU staff | original |  |
| **Working with local site staff and monitoring** | 47 | Staggered per patient payments to sites based on patient progress and data collection | original |  |
| **Working with local site staff and monitoring** | 49 | Face to face meetings with Investigators | original |  |
| **Working with local site staff and monitoring** | 50 | Teleconference meetings with Investigators | original |  |
| **Working with local site staff and monitoring** | 53 | A timeline reminder of participant visits for sites | original |  |
| **Questionnaire specific strategies** | 55 | Enhanced cover letter | original |  |
| **Questionnaire specific strategies** | 56 | Total design method, *e.g. Dillman 1978, a specific approach to maximise questionnaire response rates that utilises cover letters, reminders and resending questionnaires etc.* | original |  |
| **Questionnaire specific strategies** | 57 | Type of post used *e.g. priority, standard or recorded post* | original |  |
| **Questionnaire specific strategies** | 58 | Personal touch *e.g. handwritten letter or addition of post it notes* | original |  |
| **Questionnaire specific strategies** | 59 | Inclusion of prepaid envelope | original |  |

**Table S2: Retention strategies within the initial CTU survey but not included in the subsequent Delphi survey.**

| **Category** | **Retention Strategy** | **Included in Delphi survey** | **Reason** |
| --- | --- | --- | --- |
| Incentives | Charity Donation | n | CTUs did not appear to use this strategy and it was felt there was overlap with gifts for participants. |
| Incentives | National Lottery ticket | n | CTUs did not appear to use this strategy and it was felt there was overlap with gifts for participants. |

## Survey of HTA funded trials: Result tables

**Table S3: Respondents to the survey of HTA funded trials**

| **Respondent** | **No of trials**  **n=50** |
| --- | --- |
| Chief Investigator | 25 (50%) |
| Trial Manager | 15 (30%) |
| Other trial staff (e.g. Statistician or researcher), or combination of staff | 8 (16%) |
| Blank | 2 (4%) |
| **Total** | **50** |

**Table S4: Contextual data extracted from protocols and survey responses**

| **Health Category^1^** | **No. of trials n=50 (%)** |
| --- | --- |
| Mental health | 7 (14%) |
| Injuries | 5 (10%) |
| Oral and gastro | 5 (10%) |
| Infection | 4 (8%) |
| Renal | 4 (8%) |
| Neurological | 3 (6%) |
| Reproduction | 3 (6%) |
| Stroke | 3 (6%) |
| Blood | 2 (4%) |
| Cardiovascular | 2 (4%) |
| Respiratory | 2 (4%) |
| Cancer | 1 (2%) |
| Congenital | 1 (2%) |
| Ear | 1 (2%) |
| Eye | 1 (2%) |
| Generic | 1 (2%) |
| Inflammatory | 1 (2%) |
| Metabolic | 1 (2%) |
| Musculoskeletal | 1 (2%) |
| Skin | 1 (2%) |
| Other | 1 (2%) |
| **Recruitment status at time of survey^2^** |  |
| Closed | 34 (68%) |
| Open | 16 (32%) |
| **Intervention Type^1^** |  |
| Pharmacological | 15 (30%) |
| Medical device | 11 (22%) |
| Surgical | 5 (10%) |
| Behavioural or educational | 5 (10%) |
| Physical | 2 (4%) |
| Other | 12 (24%) |
| **Sample size^1^** |  |
| Under 100 | 0 (0%) |
| 101 to 500 | 26 (52%) |
| 501 to 1,000 | 12 (24%) |
| 1,001 to 5,000 | 9 (18%) |
| 5,001 to 10,000 | 2 (4%) |
| 10,001 and above | 1 (2%) |
| **Reporting of outcomes^1^** |  |
| Patient (and/ or carer) | 22 (44%) |
| Clinician | 19 (38%) |
| Both^3^ | 4 (8%) |
| Lab | 4 (8%) |
| Other | 1 (2%) |

^1^ Data extracted from protocols. ^2^ Data supplied by survey respondents. ^3^The outcome measurement requires both clinician and patient involvement. For example the patient reports health incidence, abstinence or occurrence of endpoint or hospitalisation that is then confirmed by clinical measurements and/ or hospital records.

**Table S5: Anticipated causes of missing data at trial design (Question 5)**

| **Anticipated causes of missing data** | **No of trials (%) n=39** |
| --- | --- |
| Factors specific to the patient population e.g. high mortality, highly mobile | 19 (49%) |
| Patients not returned data (incl. questionnaires) | 16 (41%) |
| Losing contact with patients | 12 (31%) |
| Missed measurements by clinical staff | 7 (18%) |
| Patients not attending a visit/ clinic | 7 (18%) |
| Other | 6 (15%) |
| Concerns about patient burden | 5 (13%) |
| Patient deaths | 3 (8%) |
| Patient outcomes other than death preventing measurement e.g. coma, too ill to complete measures | 3 (8%) |
| Withdrawal (Patient or clinician initiated) | 2 (5%) |
| Data not provided by clinical staff | 2 (5%) |
| Technology problems | 1 (3%) |
| Lab Problems | 0 (0%) |

***Notes:*** *The 39 respondents aware of retention issues at trial design they were given the opportunity to describe up to five areas of concern using free text fields. Responses were initially categorised using the causes of missing data used within question 11. Additional categories of patient burden and patient population were included in response to survey answers. Patient and clinician initiated withdrawal categories were combined as answers often didn’t distinguish between the two. Statements were analysed and all anticipated causes relating to the statements were recorded. e.g. ‘we are working with a population that is very frail and has high mortality’ was recorded as both a ‘factors specific to the patient population’ and ‘patient deaths’. Each anticipated cause could only be recorded once for each trial but a trial could report multiple causes.*

**Table S6: Differences between the observed and anticipated levels of missing data (Question 8)**

|  | **No of trials (%)**  **n=50** |
| --- | --- |
| As expected | 33 (66%) |
| Higher than expected | 7 (14%) |
| Lower than expected | 5 (10%) |
| Much lower than anticipated | 3 (6%) |
| Other (unknown) | 2 (4%) |
| Much higher than expected | 0 (0%) |
| **Total** | **50** |

**Table S7: Effective practices for mitigating missing data recommended from trialists (Question 13)**

| **Strategy** | **No of trials (%)**  **n= 50** |
| --- | --- |
| Monitoring (procedures and systems for monitoring data return and following up outstanding data) | 25 (50%) |
| Good site relationship/ regular contact with sites to ensure buy in | 15 (30%) |
| Site training (Initiation training and triggered training) | 11 (22%) |
| Multiple methods of data collection | 10 (20%) |
| Well-chosen measures and outcomes | 6 (12%) |
| Good patient relationship/ regular contact/ direct contact with patient | 5 (10%) |
| Incentives (general, money, prize draw) | 5 (10%) |
| Newsletters (2) | 4 (8%) |
| Simple and piloted CRFs | 4 (8%) |
| Data collection scheduled with routine care (23) | 3 (6%) |
| Staff persistence in collecting data | 3 (6%) |
| Collecting multiple contact details (4) | 3 (6%) |
| Emphasising the importance of data with the patients | 3 (6%) |
| Use of routinely collected data (27)/ follow up through patient notes | 3 (6%) |
| Taking contact details of friend/ family member (5) | 2 (4%) |
| Maintaining contact (unknown whether site or patient) | 2 (4%) |
| Simple trial design | 2 (4%) |
| Contacting patient multiple times/ different times of the week | 2 (4%) |
| Reminders (13 and 10) | 2 (4%) |
| Resources for data collection ( incl. Get UKCRN badge for resource for data return) | 2 (4%) |
| Clarity around withdrawal definition | 1 (2%) |
| Reimburse expenses | 1 (2%) |
| Method of analysis (AUC) | 1 (2%) |
| Central data return | 1 (2%) |
| Unknown | 1 (2%) |
| Data collection structure for event data | 1 (2%) |
| Phone interviews | 1 (2%) |
| Involvement of CI to help collect data | 1 (2%) |
| Internal team meeting | 1 (2%) |
| Prioritisation within trial to mitigate missing data | 1 (2%) |
| Prioritising primary outcome | 1 (2%) |
| Dedicated data staff | 1 (2%) |
| Run in period | 1 (2%) |
| Short follow up | 1 (2%) |
| Visit reminders for sites | 1 (2%) |

***Notes:*** *Although respondents were asked for three free text responses answers frequently contained more than three suggestions. We summarised all responses given and where possible aligned these with the strategies listed in the CTU survey. Additional categories such as monitoring were added where needed. Categories were reviewed and similar interventions were combined****.***

## CTU Survey: Results Tables

**Table S8: Frequency of CTUs usually adjusting their sample size for missing data (Question 2)**

|  | | **No. of CTUs**  **n=33** | **Percentage** | **Valid Percentage** |
| --- | --- | --- | --- | --- |
| Valid | No | 3 | 9.1 | 9.4 |
|  | Yes | 29 | 87.9 | 90.6 |
|  | Total | 32 | 97.0 | 100.0 |
| Missing answer | System | 1 | 3.0 |  |
| **Total** | | **33** | **100.0** |  |

**Table S9: What informs the level of attrition adjustment in the sample size calculation (Question 2)**

|  | **No of CTUs (%)**  **n=29** |
| --- | --- |
| Evidence from Literature reviews of similar trials | 20 (69%) |
| The nature of the patient population | 8 (28%) |
| Past experience | 7 (24%) |
| The type of source data/ outcome measure | 6 (21%) |
| Pilot data | 4 (14%) |
| Other | 4 (14%) |
| Length of follow up | 2 (7%) |
| Input from the Chief investigator to agree likely attrition | 2 (7%) |
| Best estimate | 1 (3%) |

***Notes:*** *Free text responses often contained more than one justification. All answers provided were categorised.*
